# Duration-dependent effects of dry cupping on autonomic modulation and trapezius tissue compliance in smartphone users: a dose-finding study

**DOI:** 10.3389/fphys.2026.1812427

**Published:** 2026-08-03

**Authors:** Chien-Liang Chen, Ioannis Manousakas, Jing-Shia Tang

**Affiliations:** 1 Department of Physical Therapy, I-Shou University, Kaohsiung, Taiwan; 2 Department of Biomedical Engineering, I-Shou University, Kaohsiung, Taiwan; 3 Department of Nursing, Chung-Hwa University of Medical Technology, Tainan, Taiwan

**Keywords:** autonomic modulation, dry cupping, heart rate variability, pressure-duration effects, tissue compliance, trapezius muscle

## Abstract

**Background:**

Prolonged smartphone use is associated with forward head posture, increased trapezius stiffness, and altered autonomic nervous system regulation. Dry cupping applies localized negative pressure-mediated mechanical stimulation that may influence myofascial properties and autonomic activity; however, its interactive effects of pressure and duration remain unclear. This study investigated the effects of different pressure and duration combinations of dry cupping on autonomic modulation indexed by heart rate variability (HRV) and trapezius tissue compliance in smartphone users.

**Methods:**

Thirty-two university students were classified into the smartphone addiction and control groups. A counterbalanced crossover design was implemented, in which each participant received four cupping protocols: ˗300 mmHg or ˗450 mmHg applied for 5 or 15 min. HRV, trapezius soft tissue compliance measured using a real-time monitoring system, and functional questionnaires were assessed before and after each intervention.

**Results:**

HRV spectral indices indicated reduced sympathetic activity and did not reach statistical significance for parasympathetic activity (p=0.062) following cupping. Compared to 5-min sessions, 15-min protocols significantly increased total power as well as both low- and high-frequency components. Trapezius tissue compliance showed the highest improvement after ˗300 mmHg applied for 15 min. Functional disability scores remained unchanged.

**Conclusion:**

In this subclinical population, dry cupping modulated autonomic activity and improved trapezius tissue compliance in a duration-dependent manner, with 15-minute applications generally producing greater effects than 5-minute applications. Treatment duration exerted a greater influence than pressure intensity, with moderate pressure sustained for 15 min producing the most pronounced autonomic and biomechanical responses.

## 1 Introduction

The widespread use of smartphones has increased dependence on these devices for occupational and leisure activities, and excessive use is associated with various health concerns (Alwazzeh et al., 2024). Prolonged smartphone use is associated with poor posture, including forward head posture and rounded shoulders, which may contribute to neck and shoulder pain and stiffness (Akodu et al., 2018; Torkamani et al., 2023). Separately, excessive smartphone use has been linked to alterations in autonomic nervous system (ANS) regulation (Nose et al., 2017; Chin et al., 2024). Smartphone addiction is strongly associated with musculoskeletal discomfort, with addicted individuals reporting more severe neck and shoulder symptoms than non-addicted users (Eitivipart et al., 2018). Given the rising prevalence of smartphone addiction, strategies targeting posture-related musculoskeletal dysfunction and associated physiological alterations are warranted. Mechanical interventions that modulate localized tissue deformation and neurovascular responses may provide insight into the mechanisms underlying such dysfunction. Negative pressure–based stimulation represents a controllable form of mechanical loading capable of inducing tissue stretch and vascular modulation; however, its quantitative pressure–duration effects on autonomic activity and soft tissue compliance remain insufficiently characterized.

Dry cupping applies localized subatmospheric pressure to the skin and underlying tissues, producing transient mechanical deformation that may influence myofascial stiffness and regional perfusion. Previous studies have reported acute changes in circulation, muscle relaxation, pain relief, activation of the heme oxygenase-1 system, and antioxidant and anti-inflammatory effects (Lowe, 2017; Al-Bedah et al., 2019). Cupping has also been shown to influence ANS activity (Arslan et al., 2014; Tang et al., 2019), potentially contributing to restoration of sympathovagal balance (El Ghazawi et al., 2025), and has been reported as effective for chronic neck pain and related myofascial pain syndromes (Lauche et al., 2011; Chiu et al., 2020; Stephens et al., 2020). Despite these findings, the physiological mechanisms underlying cupping-induced responses remain incompletely understood, and the optimal dosage has not been established (Lowe, 2017; Aboushanab and AlSanad, 2018). Importantly, key dosing parameters—including pressure intensity and application duration—vary substantially across protocols, limiting comparability and mechanistic interpretation. Therefore, a systematic comparison of different pressure and duration combinations is required to determine their influence on autonomic regulation and soft tissue mechanical properties following dry cupping.

The cupping dosage is primarily determined by the intensity of the negative pressure and application duration, which significantly influence physiological responses (Lowe, 2017; Wang et al., 2020). Clinically, pressures between ˗100 and ˗500 mmHg are commonly applied (Zhao et al., 2009; El Sayed et al., 2014). Contrastingly, extreme pressure values present challenges. Specifically, values above ˗450 mmHg may cause discomfort or soft tissue injury, whereas those below ˗150 mmHg may be insufficient to elicit measurable physiological effects (El Sayed et al., 2014; Wang et al., 2020). For example, ˗300 mmHg is more effective at increasing skin blood flow than ˗225 mmHg (Wang et al., 2020). Furthermore, both ˗300 and ˗500 mmHg have been shown to improve heart rate variability (HRV), whereas ˗100 mmHg had no significant effect (Tang et al., 2019). Therefore, ˗300 mmHg and ˗450 mmHg were selected to represent moderate- and high-pressure conditions, respectively. Although ˗500 mmHg was effective in our previous HRV study, ˗450 mmHg was chosen as the upper pressure limit in the present study to minimize participant discomfort and the potential risk of soft tissue injury during repeated crossover interventions involving four cupping sessions. Another crucial factor is treatment duration, with typical applications ranging from 5 to 15 min. Shorter durations may yield greater peak and total skin blood flow responses under the same pressure (Wang et al., 2020). Moreover, cupping at ˗300 mmHg for 10 min reduced muscle stiffness in the triceps brachii more than 5 min, although generalizability to other muscle groups or pathologically stiff tissues remains uncertain (Li et al., 2022). Overall, the interactive effects of pressure intensity and application duration on various physiological outcomes remain unclear.

Accordingly, this study systematically evaluated the effects of different pressure–duration combinations of dry cupping on autonomic activity and trapezius tissue compliance in individuals with varying levels of smartphone use, with the aim of clarifying the mechanophysiological characteristics of negative pressure stimulation and informing evidence-based dosage selection.

## 2 Methods

### 2.1 Participants

The study included 32 volunteers (22 males, 10 females; mean age, 21.7 ± 1.1 years; height, 167.5 ± 6.1 cm; weight, 65.0 ± 15.3 kg). The exclusion criteria were as follows: regular cupping therapy; cervical radiculopathy or metabolic disorders, such as diabetes; skin lesions, sensitive skin, anemia, or coagulation disorders (e.g., hemophilia, anticoagulant use) in the cupping area; and current use of medications that could influence autonomic function, pain perception, or musculoskeletal responses (e.g., muscle relaxants). Participants were instructed to maintain regular routines and avoid intense exercise or sleep deprivation on the day before the test. This study was approved by the Human Research Ethics Review Committee (NCKU HREC-E-114-0051-2). All participants provided written informed consent.

At study commencement, all participants completed the Smartphone Addiction Inventory (SPAI) and were grouped accordingly (addiction vs. control). The SPAI comprises 26 items across four domains: compulsive behavior, withdrawal, tolerance, and functional impairment. Each item is rated on a 4-point Likert scale, with a total score of 26–104. Higher scores indicate greater smartphone dependence, with a score >57 indicating problematic smartphone addiction (Lin et al., 2014). Accordingly, 19 and 13 participants were assigned to the addiction and control groups, respectively.

### 2.2 Study design

Each participant underwent four cupping interventions administered on separate days with a minimum 2-day washout period to minimize acute carryover effects. The dosages were selected based on commonly used clinical parameters (Zhao et al., 2009; El Sayed et al., 2014) and previous studies (Chen et al., 2018; Tang et al., 2019; Chiu et al., 2020). The four conditions were: (A) ˗300 mmHg for 5 min, (B) ˗450 mmHg for 5 min, (C) ˗300 mmHg for 15 min, and (D) ˗450 mmHg for 15 min. Although visible cupping marks may persist beyond the washout period, the 2-day interval was intended to reduce acute physiological carryover effects on autonomic activity, tissue compliance, and oxidative stress rather than ensure complete resolution of skin discoloration. Baseline measurements were repeated before each intervention session to ensure independent assessment of treatment effects. A counterbalanced design was applied to minimize cumulative dosage effects, generating all 24 possible intervention sequences (e.g., A→B→C→D; D→C→B→A). The first 24 participants were each assigned a unique sequence in the order of enrollment, whereas the remaining 8 were distributed across repeated sequences, ensuring that no sequence was assigned more than twice. This approach was selected to maximize treatment-order balance while maintaining feasibility within the final sample size. Because each intervention condition was represented across all ordinal positions in the initial complete counterbalancing, potential order effects were minimized despite the repeated allocation of several sequences. A fully replicated design using all 24 sequences equally was not feasible because the final sample size (n = 32) was not a multiple of the total number of possible sequences. During each session, participants were seated in a slight forward-leaning position with their elbows comfortably resting on a table to facilitate cupping of the trapezius region. All sessions were completed within a 40-day period. To ensure physiological stabilization, the participants were acclimated for 15 min in an air-conditioned laboratory (temperature: 20–24°C; relative humidity: 50–60%) before each intervention.

**Figure 1:**
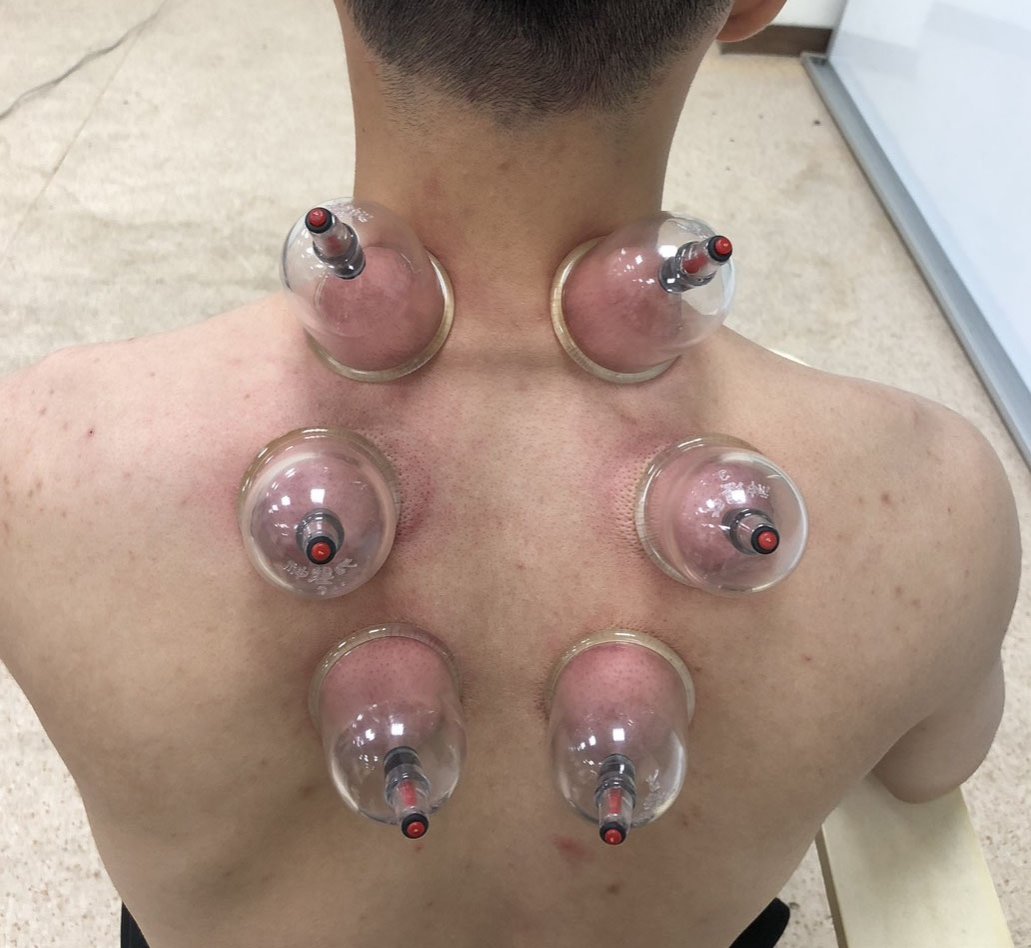
Placement of six cups bilaterally on the upper, middle, and lower trapezius according to the study protocol.

Standardization of key parameters is essential for the quantification of the cupping therapy dosage. Six cups (outer diameter: 6.5 cm; inner diameter: 5.5 cm; Shen-Nong Cupping R0416179, Income Instrument Co., Ltd., Taiwan) were applied for 5 or 15 min. The cupping locations were selected based on a previous protocol (Chiu et al., 2020), targeting the upper, middle, and lower trapezius fibers using a symmetrical six-cup configuration (**Figure 1**). As cupping effects may extend beyond the contact area (Wang et al., 2020) and influence the surrounding tissue (Li et al., 2017), a comprehensive placement across all three trapezius regions was applied to enhance therapeutic efficacy. Since smartphone addiction usually causes neck discomfort, tissue compliance measurements and blood sampling were restricted to the upper trapezius muscle on the dominant side.

### 2.3 Quantitative monitoring system

**Figure 2:**
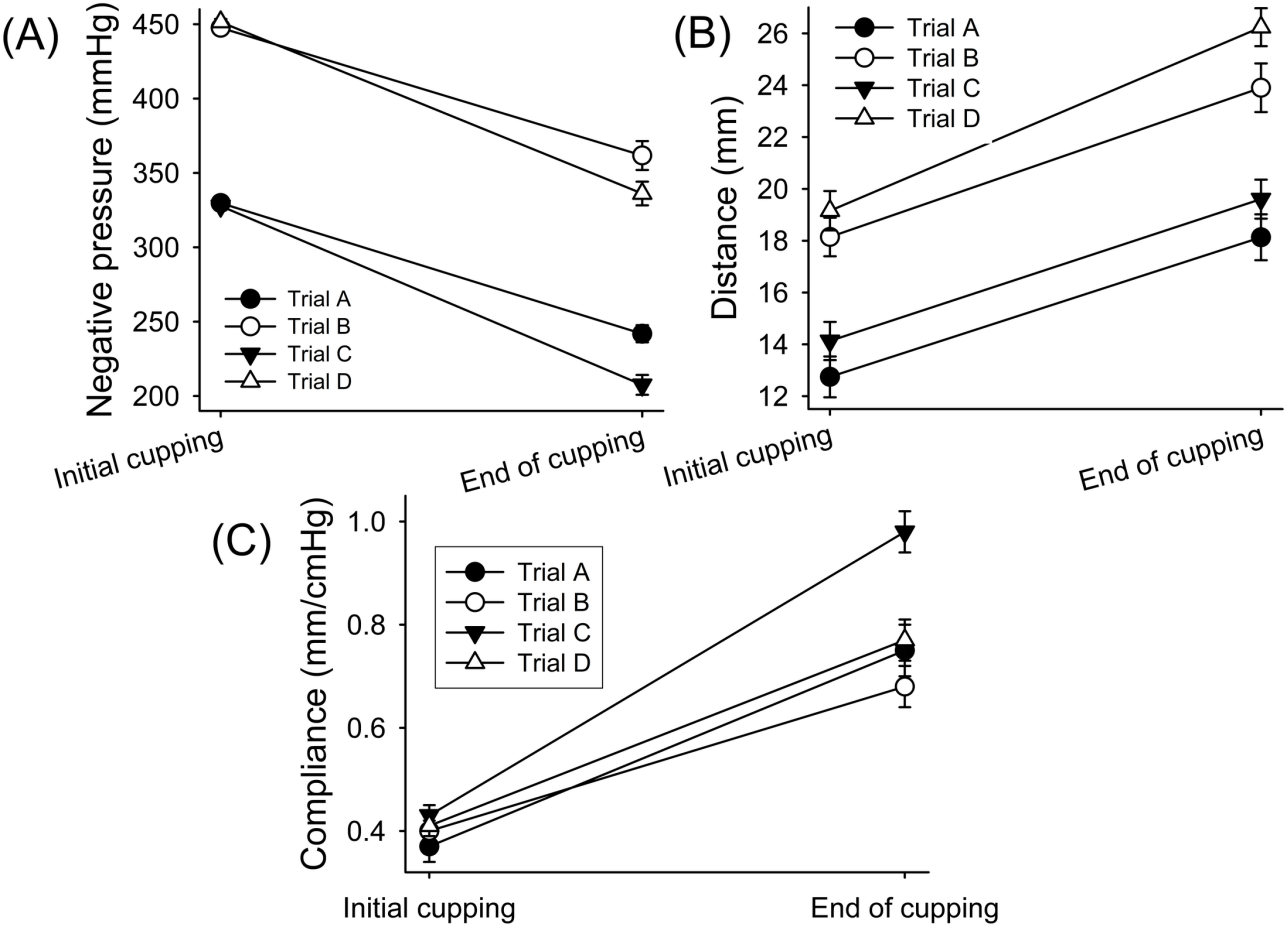
Duration-dependent effects of cupping on **(A)** vacuum pressure, **(B)** tissue displacement, and **(C)** compliance. Higher pressure increased tissue displacement, whereas moderate pressure for 15 min yielded the greatest improvement in compliance. Compliance was calculated as tissue displacement (mm) divided by the applied negative pressure (mmHg) and is presented as mm/cmHg for improved readability. Data are presented as mean ± SEM; error bars represent the standard error of the mean. Trials: A, ˗300 mmHg/5 min; B, ˗450 mmHg/5 min; C, ˗300 mmHg/15 min; D, ˗450 mmHg/15 min.

Negative pressure and soft tissue elevation within each cup were continuously monitored to evaluate soft tissue compliance, defined as the ratio of elevation height to applied pressure (mm/mmHg) (Kirsch et al., 1980). For presentation purposes, values shown in **Table 1** and **Figure 2** are expressed as mm/cmHg. Pressure sensors (PSE533, SMC, Japan), with a measurement range of +100 to ˗100 kPa, were used to meet the required operating range of 0 to ˗500 mmHg (˗67 kPa). The sensors operating at 12–24 V with an analog output of 1–5 V demonstrated high accuracy ( ± 2% FS), with a maximum error of 2.3 mmHg using a mercury sphygmomanometer.

**Table 1:** Dose-dependent effects of dry cupping on compliance of the trapezius tissue.

Dose (trials)	Parameters (Mean ± SEM) Groups	Pressure (mmHg)		Distance (mm)		Compliance (mm/cmHg)	
		Initial cupping	End of cupping	Initial cupping	End of cupping	Initial cupping	End of cupping
A	Control	330.7 ± 5.2	239.0 ± 8.9	14.39 ± 1.23	19.64 ± 1.39	0.41 ± 0.04	0.79 ± 0.07
	Addiction	328.8 ± 4.3	244.8 ± 7.3	11.10 ± 0.98	16.62 ± 1.10	0.34 ± 0.03	0.70 ± 0.06
B	Control	455.1 ± 6.7	372.9 ± 14.9	17.91 ± 1.16	23.74 ± 1.47	0.39 ± 0.02	0.65 ± 0.6
	Addiction	439.9 ± 5.5	350.4 ± 12.3	18.38 ± 0.93	24.06 ± 1.17	0.42 ± 0.02	0.72 ± 0.05
C	Control	329.5 ± 4.0	206.6 ± 10.3	14.39 ± 1.15	19.98 ± 1.18	0.44 ± 0.4	1.02 ± 0.7
	Addiction	326.0 ± 3.3	208.4 ± 8.5	13.87 ± 0.92	19.23 ± 0.94	0.43 ± 0.03	0.93 ± 0.05
D	Control	455.5 ± 3.2	331.7 ± 12.3	17.92 ± 1.19	25.9 ± 1.15	0.37 ± 0.3	0.72 ± 0.06
	Addiction	447.4 ± 2.7	340.6 ± 10.2	20.39 ± 0.95	27.39 ± 0.91	0.46 ± 0.02	0.83 ± 0.05
Time		<0.001** (612.884)		<0.001** (519.848)		<0.001** (374.135)	
Time × group		0.499 (0.468)		0.903 (0.015)		0.899 (0.016)	
Time × trial		<0.001** (7.365)		0.043* (2.837)		<0.001** (15.475)	

Doses: A, ˗300 mmHg + 5 min; B, ˗450 mmHg + 5 min; C, ˗300 mmHg + 15 min; D, ˗450 mmHg + 15 min.

Compliance values are expressed as mm/cmHg. Original values calculated in mm/mmHg were converted by multiplied by 10 (1 cmHg = 10 mmHg) for improved readability.

Data are presented as mean ± SEM; *p < 0.05, **p < 0.01.

For noninvasive assessment of tissue displacement, time-of-flight (ToF) optical sensors (VL6180X, STMicroelectronics, Switzerland) were employed because of their compact size, cost-effectiveness, and range (0–10 cm) independent of reflectivity. Precision tests using a z-axis sliding platform yielded a maximum standard deviation of 1.6 mm and an absolute error of 2 mm. To ensure stability and environmental protection, the sensors were enclosed in a custom resin-printed housing with glass windows and IR barriers.

All sensors were integrated into a custom-built control unit comprising an Arduino Nano microcontroller, an LCD screen for real-time display, a DC–DC converter, and an external power supply. The MATLAB program (MATLAB, Release 2014b, The MathWorks, Inc., Natick, MA, USA) enabled data communication, visualization (pressure and elevation over time), and CSV export. Pressure sensors were mounted via threaded ports, and ToF beam paths were aligned through drilled openings using a handheld infrared viewer (ElectroViewer 7215; Electrophysics Corporation, Fairfield, NJ, USA). The sensors were secured using an epoxy resin. The detailed specifications have been previously described (Chiu et al., 2020).

### 2.4 Outcome measures

#### 2.4.1 Functional assessment of the neck and upper extremities

The Neck Disability Index (NDI) is a self-reported questionnaire for assessing neck pain and its impact on daily activities (Vernon and Mior, 1991). Its Chinese version, validated by Wu et al. (2010) (Wu et al., 2010), exhibits high internal consistency (Cronbach’s α=0.89) and test-retest reliability (r=0.95). It comprises 10 items (pain intensity, personal care, lifting, reading, headaches, concentration, work, driving, sleep, and recreation), scoring from 0 (no disability) to 5 (complete disability), with in a total score of 0–50. Higher scores indicate greater functional impairment. Based on the NDI scores, disability severity was classified as follows: 0–4 (no disability), 5–14 (mild), 15–24 (moderate), 25–34 (severe), and ≥35 (complete perceived disability).

The validated Chinese version of the Disabilities of the Arm, Shoulder, and Hand (DASH) questionnaire was used to evaluate upper extremity function (Liang et al., 2004). The DASH comprises 30 items that assess physical function, pain, and symptoms. Each item is rated on a 5-point Likert scale, resulting in a total score of 0–100, with higher scores indicating more severe disability. The Chinese version of this questionnaire exhibits good internal consistency (Cronbach’s α=0.96) and test-retest reliability (intraclass correlation coefficient=0.9).

#### 2.4.2 Soft tissue compliance assessment

Soft tissue compliance in the dominant side of the upper trapezius was assessed using a quantitative dry-cupping monitoring system. Higher compliance indicates softer and more flexible tissues, whereas lower compliance suggests greater stiffness. Compared with healthy individuals, those with myofascial pain syndrome have significantly lower trapezius compliance (Chiu et al., 2020). Assessing the relationship between tissue elevation and pressure decay during cupping (5 or 15 min) is crucial, as compliance varies across individuals and can be influenced by various pathologies. This allows real-time monitoring of the influence of different cupping dosages, based on pressure and duration, on the soft tissue response.

#### 2.4.3 Oxidative stress assessment

Blood oxidative stress levels were assessed using a free oxygen radical test (FORT) analyzer (Callegari, Catellani group, Parma, Italy) (Pavlatou et al., 2009). As obtaining sufficient blood samples for FORT analysis via lancet puncture was difficult because of the substantial tissue swelling immediately after cupping, capillary whole blood samples (20 μL for FORT) were collected from a site 1 cm outside the cupping area on the upper trapezius before and after cupping intervention. The samples were immediately processed at room temperature by mixing with the reagents, followed by centrifugation at 5000 rpm (2000 g) for 1 min. The CR3000 analyzer was used to measure absorbance at 505 nm and 37°C to yield quantitative FORT values. The intra- and inter-assay coefficients of variation were <5% and <7%, respectively. The FORT reflects reactive oxygen species (ROS) levels, with the normal range being ≤2.3 mmol/L H_2_O_2_. The ROS level is a validated oxidative stress marker in metabolic disorders, such as type 2 diabetes and obesity (Găman et al., 2020). Oxidative stress was included as an exploratory physiological marker in this study.

#### 2.4.4 HRV assessment

To evaluate the response of the ANS to different cupping dosages, HRV was continuously monitored using a portable electrocardiography (ECG) recorder (TD3; Taiwan Telemedicine Device Company). ECG signals (lead II) were recorded for 5 min and used to analyze variability in the R interval. Frequency–domain HRV indices, including total power (TP), low frequency (LF), high frequency (HF), and LF/HF ratio, were derived from 5-min recordings using Fast Fourier Transform analysis. LF% and HF% were calculated as LF/TP × 100 and HF/TP × 100, respectively, following the methodology of the Kuo HRV Analysis Software (Kuo et al., 1999). Unlike the normalized units recommended by the Task Force (Task Force of the European Society of Cardiology and the North American Society of Pacing and Electrophysiology, 1996), TP − VLF was not used as the denominator. Therefore, LF% and HF% were not expected to sum to 100%, with the remaining proportion reflecting VLF and other spectral components. TP reflects overall autonomic activity; LF power reflects both sympathetic and parasympathetic modulation; HF power primarily reflects parasympathetic activity, LF% is associated with sympathetic activity, whereas HF% is associated with sympathetic inhibition; and the LF/HF ratio is commonly used to assess sympathovagal balance. Further details regarding the HRV acquisition and processing are previously described (Chen et al., 2015; Tang et al., 2019).

### 2.5 Statistical analysis

Data distribution normality was assessed using the Shapiro–Wilk test prior to parametric analysis, confirming that all primary outcome variables met the assumptions required for repeated-measures analysis of variance (RM-ANOVA). RM-ANOVA was conducted to examine the effects of dry cupping on the subjective and objective physiological responses. Subjective outcomes included the pre- and post-intervention NDI and DASH scores. Objective outcomes included immediate pre- and post-intervention measurements of soft tissue compliance, oxidative stress, and HRV parameters. A 2×2 (group×time) RM-ANOVA was used to test for interaction effects between the groups and time. Further, a 4×2 (trial×time) RM-ANOVA was used to compare the interaction effects across the four cupping dosages. For HRV outcomes, an additional supplementary 2 × 2 × 2 RM-ANOVA was conducted with pressure (−300 vs. −450 mmHg), duration (5 vs. 15 min), and time (pre- vs. post-intervention) as within-subject factors to directly examine the independent and interactive effects of pressure and duration. All statistical analyses were performed using PASW Statistics software (version 23.0; SPSS Inc., Chicago, IL, USA). Statistical significance was set at p<0.05, with trends being considered at 0.05<p<0.09.

The adequacy of the sample size was assessed using an *a priori* power analysis using G*Power (version 3.1.9.4, Heinrich-Heine-Universität Düsseldorf, Germany), based on a RM-ANOVA with an assumed effect size of 0.60 (Nasb et al., 2020), power of 0.80, and α=0.05. Accordingly, the minimum required sample size was 24 participants, and the final sample (N=32) satisfied the statistical requirements. The study was powered for primary physiological outcomes. Because several HRV indices were derived from the same physiological signal and are therefore not statistically independent, HRV findings were interpreted collectively as complementary indicators of autonomic modulation. Given the exploratory nature of this dose-finding study, secondary physiological and functional outcomes were interpreted cautiously.

## 3 Results

### 3.1 Cupping dose effects on neck and upper extremities disability scores

No significant difference was observed in the NDI (p=0.579) or DASH (p=0.783) scores across the four cupping trials (**Table 2**). No significant between-group difference was observed in the NDI (p=0.564) or DASH (p=0.528) scores. No significant interaction effect was observed between the group and cupping dose (trial-by-time interaction, p>0.05).

**Table 2:** Self-perceived neck and upper limb disabilities across cupping dosages.

Self-perception scale	Groups Trials	Control (N = 13)	Smartphone addiction (N = 19)	p (F) value		
				Group	Trial	Trial × Group
NDI	A	3.692 ± 1.029	2.611 ± 0.874	0.564	0.579	0.185
	B	3.154 ± 0.780	2.111 ± 0.663	(0.341)	(0.562)	(1.733)
	C	3.077 ± 0.793	1.556 ± 0.674			
	D	2.231 ± 1.322	3.444 ± 1.123			
DASH	A	1.059 ± 0.554	1.858 ± 0.471	0.528	0.783	0.163
	B	1.507 ± 0.639	1.682 ± 0.543	(0.407)	(0.283)	(1.830)
	C	1.627 ± 0.427	0.863 ± 0.363			
	D	1.003 ± 0.735	2.224 ± .625			

Trials: A, ˗300 mmHg + 5 min; B, ˗450 mmHg + 5 min; C, ˗300 mmHg + 15 min; D, ˗450 mmHg + 15 min.

Data are presented as mean ± standard error of the mean (SEM).

DASH, Disability of Arm, Shoulder and Hand; NDI, Neck Disability Index.

No significant correlation was observed between SPAI and NDI (r < 0.001, p=0.999) or between SPAI and DASH scores (r=0.174, p=0.340). However, a moderate positive correlation was observed between the NDI and DASH scores (r=0.385, p=0.029). The mean NDI and DASH scores were 2.72 and 1.78, respectively (**Table 3**), which are low and indicated minimal neck or upper limb disability. These findings are consistent with those shown in **Table 2**, indicating no significant between-group differences in the NDI (p=0.564) and DASH (p=0.528) scores.

**Table 3:** Pearson’s correlations among the SPAI, NDI, and DASH scores (N = 32).

Variables	Items Mean (SD)	Statistical Values	SPAI	NDI	DASH
SPAI	58.66 (16.03)	*r*	−		
		p-value			
NDI	2.72 (2.79)	*r*	0.000	−	
		p-value	0.999		
DASH	1.78 (2.16)	*r*	0.174	0.385	−
		p-value	0.340	0.029*	

Data are presented as mean ± standard deviation (SD). *p< 0.05.

r, correlation coefficient; SPAI, smartphone addiction inventory; NDI, neck disability index; DASH, arm, shoulder, and hand disability.

### 3.2 Between-group comparisons of the cupping dose effects on blood oxidative stress

Blood oxidative stress (FORT) did not significantly differ across the cupping dosages (trial effect and trial-by-time interaction, p>0.05; **Table 4**). No significant between-group difference was observed in the oxidative stress levels (trial-by-group and time-by-group interactions, p>0.05). No significant increase was observed in oxidative stress at 5–15 min post cupping (time effect, p>0.05). These findings do not permit conclusions regarding localized or delayed oxidative stress responses.

**Table 4:** Effects of the cupping dose on FORT values at the edge of the cupping site.

Dose (trial)	Parameters Groups	FORT U		H_2_O_2_ (mmol/L)	
		Pre-cupping	Post-cupping	Pre-cupping	Post-cupping
A	Control	213.2 ± 25.5	206.8 ± 23.6	1.62 ± 0.19	1.57 ± 0.18
	Addiction	258.1 ± 21.1	261.9 ± 19.5	1.96 ± 0.16	1.99 ± 0.15
B	Control	225.7 ± 26.7	214.8 ± 27.9	1.72 ± 0.20	1.63 ± 0.21
	Addiction	267.6 ± 22.1	267.2 ± 23.1	2.04 ± 0.17	2.03 ± 0.18
C	Control	210.5 ± 24.1	207.2 ± 22.2	1.60 ± 0.18	1.58 ± 0.17
	Addiction	262.6 ± 19.9	263.3 ± 18.4	2.00 ± 0.15	2.00 ± 0.14
D	Control	220.8 ± 25.1	226.8 ± 22.5	1.67 ± 0.19	1.73 ± 0.17
	Addiction	258.6 ± 20.8	257.2 ± 18.6	1.97 ± 0.16	1.96 ± 0.14
p (F) value					
Trial effect		0.530 (0.610)		0.529 (0.612)	
Trial × group		0.518 (0.634)		0.512 (0.645)	
Time effect		0.581 (0.311)		0.589 (0.298)	
Time × group		0.413 (0.690)		0.418 (0.674)	
Trial × time		0.594 (0.576)		0.589 (0.583)	

Doses: A, ˗300 mmHg + 5 min; B, ˗450 mmHg + 5 min; C, ˗300 mmHg + 15 min; D, ˗450 mmHg + 15 min.

Data are presented as mean ± SEM.

FORT, Free Oxygen Radical Test.

### 3.3 Effects of cupping dose on tissue mechanical properties

A pressure-duration interaction was observed across the four cupping conditions (**Figure 2**). The overall cupping process influenced soft tissue behavior by gradually reducing the negative pressure inside the cup, increasing tissue displacement, and consequently enhancing tissue compliance over time (time effect, p<0.001; **Table 1**). No significant between-group difference was observed in cupping pressure, tissue lifting distance, or tissue compliance (time-by-group interaction, p>0.05). Further analysis of pressure dynamics across different cupping doses revealed that groups with longer cupping durations (15 min in Trials C and D) exhibited a greater decline in negative pressure over time than their shorter-duration counterparts (5 min in Trials A and B), despite the same initial pressure (time-by-trial interaction, p<0.001; **Figure 2A**). Regarding tissue displacement, groups with higher negative pressure (Trials B and D) showed significantly greater tissue lifting than those with lower pressure (Trials A and C), indicating a significant time-by-trial interaction (p=0.043; **Figure 2B**). Regarding tissue compliance, Trial C (−300 mmHg for 15 min) produced the greatest increase in tissue compliance (p < 0.001; **Figure 2C**), whereas the highest-pressure condition (−450 mmHg for 15 min; Trial D) did not result in further improvement. These findings suggest a non-linear pressure–duration relationship, with moderate pressure combined with longer application duration producing the most favorable response among the tested conditions.

### 3.4 Cupping dose effects on HRV in smartphone-addicted and control groups

**Figure 3:**
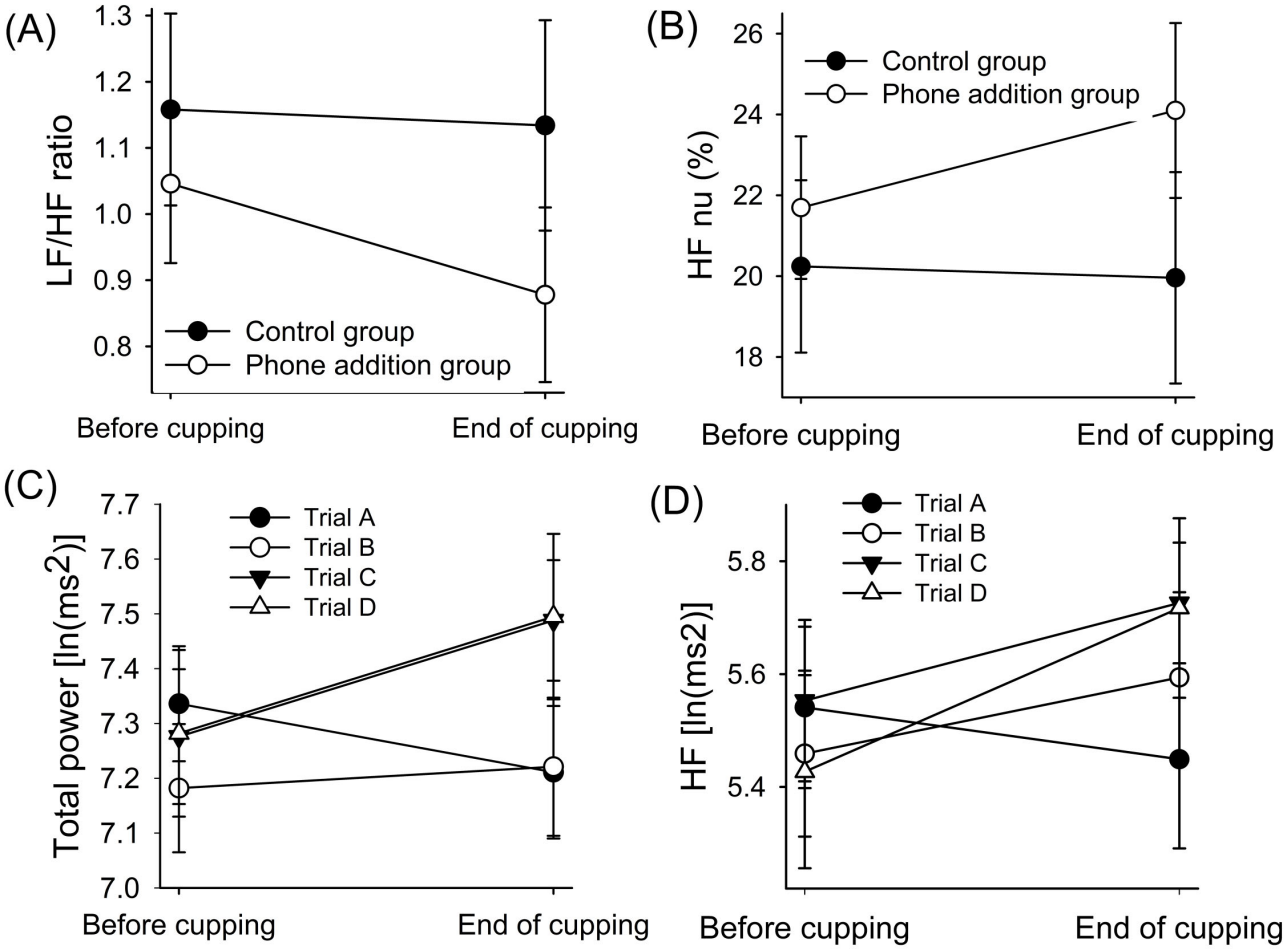
Effects of cupping on HRV by group and dosage. **(A)** Trend toward reduced sympathetic dominance in the smartphone addiction group. **(B)** Trend toward increased sympathetic inhibition. **(C)** Longer-duration cupping (Trials C and D) increased total autonomic activity. **(D)** Parasympathetic activity increased following 15-min cupping. Trials: A, ˗300 mmHg/5 min; B, ˗450 mmHg/5 min; C, ˗300 mmHg/15 min; D, ˗450 mmHg/15 min.

HRV parameters demonstrated distinct responses across the four cupping conditions, with longer-duration protocols generally showing greater changes than shorter-duration protocols (**Figure 3**). Overall, cupping significantly reduced sympathetic nervous activity (**Table 5**), as indicated by a decrease in the LF/HF ratio (p=0.028) and LF% (p=0.022), showing a trend toward increased parasympathetic activity (HF, p=0.062). Compared with the control group, the smartphone addiction group showed a tendency of post-intervention reduction in sympathetic activity (time-by-group interaction, LF/HF ratio, p=0.096; **Figure 3A**). A trend toward greater sympathetic inhibition, as indicated by increased HF% (p=0.072; **Figure 3B**), was also observed. Regarding the effects of the cupping dose, longer-duration trials (15 min; Trials C and D) yielded significantly greater increases in autonomic total power (TP; time-by-trial interaction, p=0.010; **Figure 3C**), as well as improved sympathetic–parasympathetic modulation (LF; p=0.011), compared with shorter-duration trials. Moreover, longer cupping durations significantly enhanced parasympathetic activity (HF; p=0.028; **Figure 3D**). An additional factorial RM-ANOVA showed no significant pressure × duration × time interaction for any HRV parameters (**Supplementary Table 1**). The pattern of HRV responses remained consistent with the primary trial × time (4 × 2) analysis, in which longer application duration was associated with greater changes than shorter application duration.

**Table 5:** Effects of cupping dose on heart rate variability parameters.

HRV	Group	Control (N = 13)		Addiction (N = 19)		p (F) value		
	Trials	Before cupping	The end of cupping	Before cupping	The end of cupping	Time	Time × Group	Time × Trial
TP [ln(ms^2^)]	A	7.10 ± 0.16	6.95 ± 0.19	7.58 ± 0.13	7.48 ± 0.15	0.202	0.794	0.010*
	B	7.01 ± 0.18	7.01 ± 0.19	7.36 ± 0.15	7.43 ± 0.16	(1.704)	(0.069)	(3.982)
	C	7.25 ± 0.19	7.43 ± 0.17	7.30 ± 0.16	7.55 ± 0.14			
	D	7.16 ± 0.24	7.54 ± 0.23	7.41 ± 0.19	7.45 ± 0.19			
LF [ln(ms^2^)]	A	6.47 ± 0.17	6.33 ± .21	6.86 ± 0.14	6.68 ± 0.17	0.642	0.576	0.011*
	B	6.38 ± 0.19	6.34 ± 0.20	6.68 ± 0.16	6.65 ± 0.16	(0.221)	(0.319)	(3.894)
	C	6.55 ± 0.21	6.75 ± 0.18	6.56 ± 0.17	6.78 ± 0.15			
	D	6.57 ± 0.26	6.82 ± 0.24	6.71 ± 0.21	6.67 ± 0.19			
HF [ln(ms^2^)]	A	5.29 ± 0.22	5.06 ± 0.24	5.79 ± 0.18	5.84 ± 0.20	0.062#	0.606	0.028*
	B	5.27 ± 0.23	5.36 ± 0.23	5.64 ± 0.19	5.83 ± 0.19	(3.762)	(0.271)	(3.176)
	C	5.49 ± 0.22	5.66 ± 0.17	5.61 ± 0.18	5.79 ± 0.14			
	D	5.28 ± 0.26	5.63 ± 0.25	5.58 ± 0.22	5.81 ± 0.20			
LF/HF Ratio	A	1.17 ± 0.19	1.27 ± 0.22	1.07 ± 0.16	0.84 ± 0.18	0.028*	0.096#	0.219
	B	1.11 ± 0.19	0.98 ± 0.19	1.04 ± 0.16	0.82 ± 0.15	(5.325)	(2.945)	(1.504)
	C	1.06 ± 0.20	1.09 ± 0.19	0.94 ± 0.17	0.99 ± 0.16			
	D	1.30 ± 0.16	1.20 ± 0.21	1.13 ± 0.13	0.86 ± .17			
LF%	A	72.9 ± 3.7	73.6 ± 4.2	69.8 ± 3.1	66.1 ± 3.5	0.022*	0.178	0.362
	B	70.8 ± 3.9	68.8 ± 3.9	69.7 ± 3.2	65.4 ± 3.2	(5.810)	(1.905)	(1.079)
	C	71.5 ± 4.2	71.4 ± 3.6	67.5 ± 3.5	67.8 ± 3.0			
	D	75.6 ± 3.0	73.7 ± 4.2	71.9 ± 2.5	67.0 ± 3.4			
HF%	A	19.5 ± 2.8	18.8 ± 3.6	21.3 ± 2.3	25.4 ± 2.9	0.151	0.072#	0.471
	B	21.3 ± 3.0	22.1 ± 3.2	22.3 ± 2.4	24.2 ± 2.6	(2.172)	(3.481)	(0.849)
	C	21.2 ± 3.0	19.9 ± 2.7	22.9 ± 2.5	22.5 ± 2.2			
	D	19.0 ± 2.3	19.0 ± 3.7	20.3 ± 1.9	24.4 ± 3.0			

HRV, heart rate variability; TP, total power; LF, low-frequency power; HF, high-frequency power; LF/HF, LF to HF ratio; LF%, LF in normalized units.

Trials: A, ˗300 mmHg + 5 min; B, ˗450 mmHg + 5 min; C, ˗300 mmHg + 15 min; D, ˗450 mmHg + 15 min.

Data are presented as mean ± SEM; ^#^0.05 < p < 0.09 (trend of significant difference), *p < 0.05.

## 4 Discussion

The findings of this study indicated that dry cupping significantly modulates autonomic function and enhances soft tissue compliance, specifically at moderate pressure (˗300 mmHg) for 15 min. This condition yielded the greatest improvement in trapezius compliance, whereas shorter duration and higher pressure did not produce similar effects. The observed autonomic and biomechanical responses may be explained by localized negative pressure–induced tissue deformation and subsequent neurovascular modulation. Application of subatmospheric pressure likely produces controlled mechanical stretch of the skin, fascia, and underlying musculature, leading to activation of mechanoreceptors and modulation of local microcirculation (Henriksen, 1976). Mechanical deformation may influence muscle spindle sensitivity (Bewick and Banks, 2015) and afferent signaling, thereby altering central autonomic integration through somatosensory–autonomic reflex pathways (Sato and Schmidt, 1987). A longer application duration (15 min) may provide more sustained mechanical stimulation of the skin and underlying soft tissues, which could contribute to the greater improvements in parasympathetic activity and tissue compliance observed in this study. However, the physiological mechanisms underlying these responses were not directly assessed and warrant further investigation. Within the tested pressure range, longer-duration protocols were associated with greater autonomic modulation and improvements in tissue compliance than shorter-duration protocols under the tested conditions.

Furthermore, no significant between-group differences were observed, indicating comparable responses to cupping. Functional assessment scores confirmed the absence of musculoskeletal disability. The mean NDI scores were <4 in both groups, indicating no functional impairment (Vernon, 2008), with no significant group effect. Similarly, the DASH scores were 0.86–2.22 (out of 100), indicating no significant between-group difference (p=0.528). The absence of significant deficits in neck or upper limb function in the addiction group can be attributed to the young age (mean=21.72 ± 1.11 years) and overall musculoskeletal health. Additionally, the mean SPAI score in the addiction group (69 out of 104) reflected mild addiction. Therefore, no significant between-group differences were observed in the measured physiological outcomes. Under the present experimental conditions, smartphone usage status was not associated with differential autonomic or tissue compliance responses. Unlike previous reports indicating that cupping improved DASH scores in patients with myofascial pain syndrome (Chiu et al., 2020), our participants exhibited minimal functional impairment at baseline, which may have limited the ability to detect between-group differences. In addition, the study was not specifically powered to detect group-by-treatment interactions; therefore, non-significant findings should not be interpreted as evidence of equivalence between groups. However, because neither a sham nor a no-treatment control condition was included, the extent to which the observed changes can be attributed specifically to cupping therapy remains uncertain. Further longitudinal studies incorporating appropriate control conditions are warranted to clarify the potential role of cupping in health promotion and early intervention.

Prolonged poor posture induces myofascial pain and inflammation, which significantly increases oxidative stress and correspondingly decreases antioxidant capacity (Koca et al., 2014). Wet cupping may eliminate oxidants and reduce oxidative stress (Tagil et al., 2014). Additionally, dry cupping may activate the heme oxygenase-1 system, yielding antioxidant and anti-inflammatory effects (Lowe, 2017; Stephens et al., 2020). Contrastingly, the present study did not observe significant changes in oxidative stress as measured by FORT values following cupping intervention. Because blood samples were collected adjacent to the cupping site rather than directly from the stimulated tissue, localized oxidative responses may have been diluted by systemic circulation. Furthermore, oxidative stress responses may exhibit delayed temporal dynamics beyond the immediate post-intervention measurement window. Therefore, the absence of significant changes in FORT values should be interpreted cautiously. Future studies employing localized sampling techniques, such as microdialysis or interstitial fluid analysis, and incorporating extended follow-up measurements may provide a more direct assessment of localized biochemical responses following cupping therapy. Additionally, our participants did not exhibit severe neck or shoulder dysfunction, which may have reduced the likelihood of detecting substantial oxidative stress elevations.

The observed increase in tissue displacement during cupping reflects the mechanical interaction between negative pressure and superficial soft tissue structures. The compliance index derived in this study represents the macroscopic pressure–displacement behavior of tissue under negative pressure loading rather than a direct measure of intrinsic material stiffness. Tissue deformation may involve multiple processes, including viscoelastic relaxation, interstitial fluid redistribution, and microvascular responses. Therefore, the increased displacement observed in the present study should be interpreted as a composite mechanophysiological response to sustained negative pressure stimulation. This response may reflect changes in tissue deformability associated with fascial glide, fluid dynamics, and mechanoreceptor activation, rather than a pure alteration in elastic material properties. Cupping therapy has been reported to reduces muscle stiffness and pain while improving muscle flexibility and range of motion (aan et al., 2021). In this study, soft tissue compliance was quantified as tissue elevation relative to the applied negative pressure (Vernon and Mior, 1991). This measure reflects the deformation response of soft tissues to externally applied negative pressure and has previously been reported to be greater in healthy tissues than in chronically stressed or overused regions (Chiu et al., 2020). However, it should be interpreted as an indirect indicator of tissue deformability rather than a direct measure of muscle stiffness. In the present study, no significant between-group difference was observed in compliance (**Table 1**; time-by-group interaction, p=0.899), which can be attributed to the participants’ mild addiction levels and the absence of notable neck or shoulder dysfunction or inflammation-related oxidative stress. The observed pressure-duration response pattern provides insight into the underlying mechanisms. A negative pressure of ˗450 mmHg is considered excessive and may harm the soft tissue within the cupping area (Tian et al., 2007; Wang et al., 2020). Biological tissues exhibit nonlinear viscoelastic characteristics, and higher negative pressure does not necessarily produce proportionally greater physiological responses. Excessive negative pressure may induce microvascular compression or protective neuromuscular responses that limit further deformation or adaptation. Contrastingly, moderate pressure may prevent excessive tissue tension and discomfort, facilitate relaxation, and improve patient compliance. Longer application durations may sustain mechanical stimulation of cutaneous and myofascial mechanoreceptors, enhance local blood flow, and modulate autonomic function. Therefore, the superior response observed at ˗300 mmHg for 15 min should be interpreted as reflecting an optimal moderate-pressure condition within a favorable physiological loading range rather than a simple linear pressure–response relationship. Collectively, these factors may explain why the ˗300 mmHg for 15 min protocol produced the greatest improvement in tissue compliance among the tested conditions.

In our study, dry cupping significantly reduced sympathetic activity and showed a trend toward enhanced parasympathetic activation. This is consistent with previous reports that cupping modulates sympathovagal balance via β-endorphin and adrenocortical hormone release (Arslan et al., 2014; El Ghazawi et al., 2025). Furthermore, higher post-intervention autonomic responses were observed in the smartphone addiction group than in the control group (**Figures 3A, B**). This suggests that ANS modulation may precede changes in symptoms, oxidative stress, or tissue compliance. Additionally, analyses of the four cupping protocols indicated that treatment duration was more consistently associated with HRV changes than pressure intensity. Specifically, 15-min cupping significantly enhanced total power and HF components compared to 5-min cupping; contrastingly, pressure variations (˗300 vs. ˗450 mmHg) did not significantly influence HRV outcomes (**Figures 3C, D**). Although autonomic modulation appeared to be more strongly associated with treatment duration than pressure intensity, pressure-related effects and pressure–duration interactions were less robust. Therefore, the findings should be interpreted as duration-dependent responses under the tested conditions rather than evidence of a simple linear dose–response relationship. Future studies with larger samples, sham-controlled designs, and confirmatory statistical frameworks are needed to further validate these findings. Importantly, the interpretation of autonomic responses was based on the overall consistency of response patterns across multiple physiologically related HRV variables rather than on isolated statistically significant findings.

A potential placebo or nonspecific relaxation effect should be considered when interpreting autonomic outcomes, as HRV indices are sensitive to contextual influences such as expectation and passive rest. However, in our previous time-course study, minimal suction pressure (−100 mmHg), which provides limited mechanical stimulation, did not produce significant changes in HRV, whereas moderate and higher pressures (−300 and −500 mmHg) resulted in significant autonomic modulation primarily during the post-intervention recovery period rather than during active suction (Tang et al., 2019). This temporal pattern suggests that HRV responses may reflect a physiological recovery process following mechanical stimulation rather than an immediate psychophysiological effect. The similar adaptation trends observed across pressure conditions indicate that autonomic changes may arise from combined mechanophysiological and contextual influences. Future sham-controlled studies incorporating simulated or minimal suction conditions and extended observation periods are warranted to further distinguish specific physiological effects from placebo-related mechanisms.

Cupping therapy has shown dose-dependent effects on various physiological domains. First, for skin blood flow, higher pressure (˗300 vs. ˗225 mmHg) and shorter duration (5 vs. 10 min) are associated with greater peak and total blood flow (Wang et al., 2020). Second, in terms of muscle stiffness, longer application durations yield more substantial reductions (Li et al., 2022). Third, cupping at ˗100 mmHg for 15 min did not significantly affect HRV, whereas ˗300 and ˗500 mmHg significantly improved HRV outcomes (Tang et al., 2019). Our results revealed that prolonged duration, but not pressure intensity, had a significant effect on HRV.

From a clinical perspective, these findings may help inform the optimization of cupping prescriptions. Moderate pressure applied for a longer duration was associated with favorable physiological responses while minimizing discomfort, suggesting a simple and potentially applicable protocol for individuals with muscle stiffness, posture-related complaints, or autonomic imbalance.

### 4.1 Limitations

Several methodological considerations should be acknowledged. First, this study examined only pressure and duration and did not evaluate other treatment parameters, such as cup size, number of cups, or anatomical sites. Second, the intervention was limited to the trapezius region; therefore, the findings may not be generalizable to other anatomical areas, including the lumbar or lower-extremity regions. Third, most participants were young adults with generally healthy soft tissues, which may limit the applicability of the findings to older adults or individuals with musculoskeletal disorders. In addition, the study was powered to detect the primary intervention effects rather than group-by-treatment interactions, which may have limited the ability to identify small between-group differences. Consequently, the absence of significant interaction effects should not be interpreted as evidence of equivalent responses between the smartphone addiction and control groups. Fourth, oxidative stress sampling was performed adjacent to, rather than directly beneath, the cupping sites and was assessed only immediately after intervention. Therefore, the present study cannot adequately evaluate localized or delayed oxidative stress responses associated with cupping therapy. Fifth, the absence of a sham-control group limits the ability to distinguish intervention-specific physiological effects from nonspecific or time-dependent responses. Sixth, outcome assessors were not formally blinded to group allocation or intervention condition, which may have introduced measurement bias despite the use of standardized instrumentation and objective outcome measures. Seventh, the sex distribution was uneven (22 males and 10 females), which may limit the generalizability of the finding because potential sex-related differences in autonomic regulation and soft tissue responses to cupping cannot be adequately evaluated. Despite these limitations, the standardized pressure-controlled design provides a reproducible framework for quantifying cupping parameters and systematically comparing the effects of negative-pressure magnitude and application duration.

## 5 Conclusions

This study provides quantitative evidence regarding the mechanophysiological responses associated with different dry cupping protocols. Moderate negative pressure applied for 15 min elicited the most favorable autonomic and soft tissue responses, suggesting an optimal moderate-pressure condition rather than a simple linear pressure–response relationship. Collectively, these findings indicate that longer application durations were associated with greater autonomic and tissue compliance responses under the conditions tested.

## Data Availability

The original contributions presented in the study are included in the article/**Supplementary Material**. Further inquiries can be directed to the corresponding author.
