# Supplementary material for: Duration-dependent effects of dry cupping on autonomic modulation and trapezius tissue compliance in smartphone users: a dose-finding study
Source: Front Physiol. 2026 Aug 3;17:1812427. doi: 10.3389/fphys.2026.1812427 (PMC13477954; doi:10.3389/fphys.2026.1812427)
Supplement: Supplementary file 1 [file DataSheet1.pdf]

Effect of Dry Cupping on Autonomic Activity and Trapezius Compliance

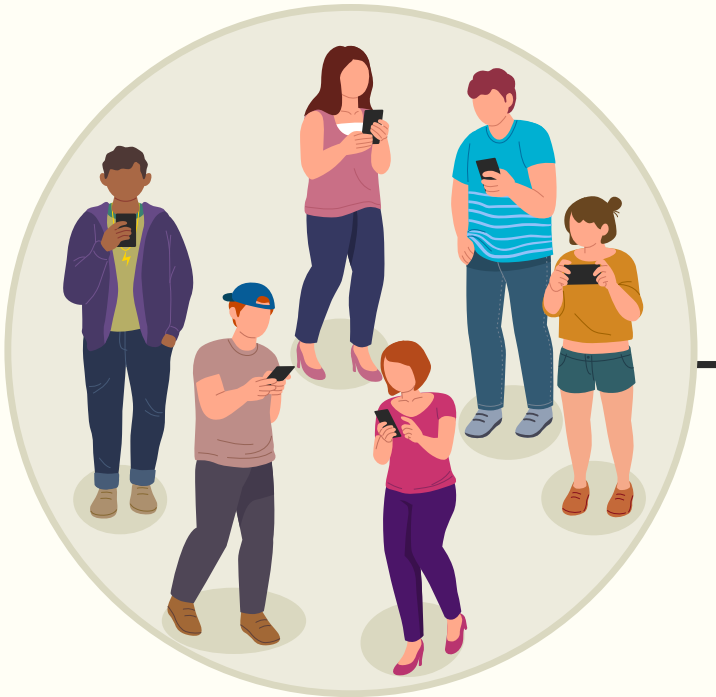

University students using smartphone (N = 32)

- Addiction group (n = 19)
  - ⚠ **Leads to trapezius muscle stiffness**
- Control group (n = 13)

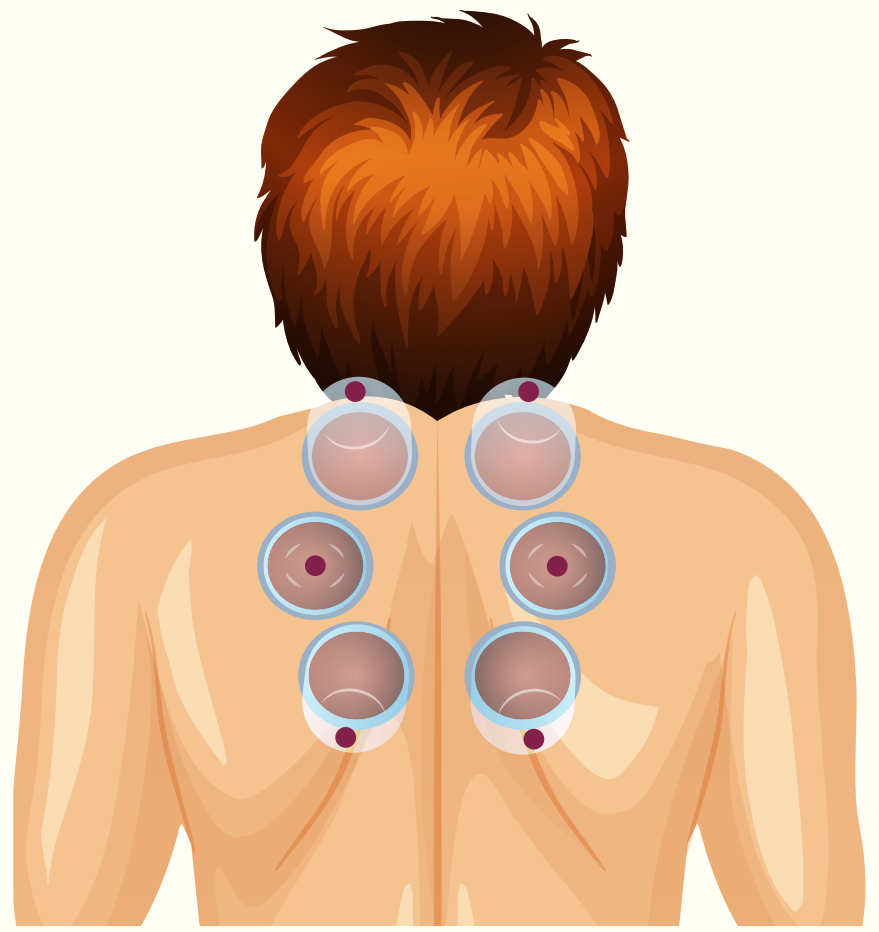

Trapezius muscle cupping

Cupping protocols

- −300 mmHg, 5 minutes
- −450 mmHg, 5 minutes
- −300 mmHg, 15 minutes
- −450 mmHg, 15 minutes

−300 mmHg and −450 mmHg refer to negative pressure applied during cupping. 5 minutes and 15 minutes refer to the time duration for cupping

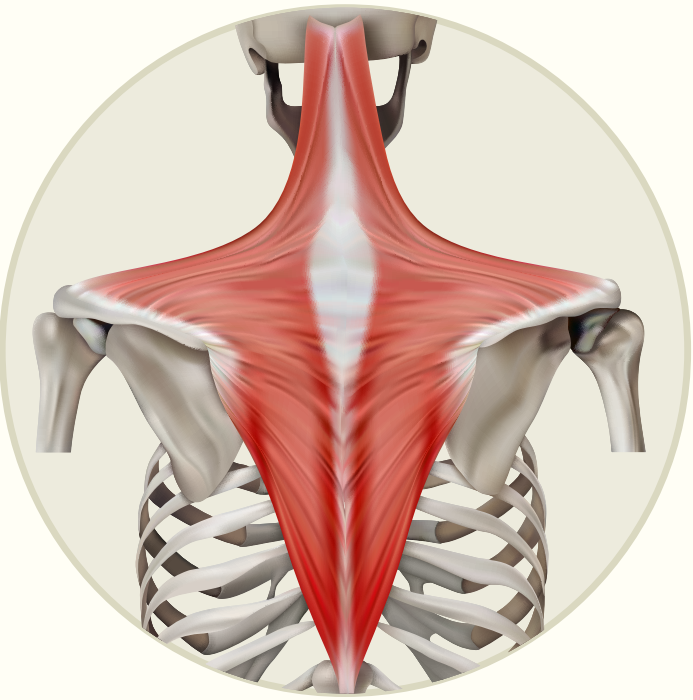

Improved tissue compliance

Longer cupping durations (15 minutes)	→	↓ Negative pressure ($p < 0.001$)
Higher negative pressure (−450 mmHg)	→	↑ Tissue lifting ($p = 0.043$)
**Cupping at −300 mmHg for 15 minutes**	→ ($p = 0.001$)	↑ **Tissue compliance**

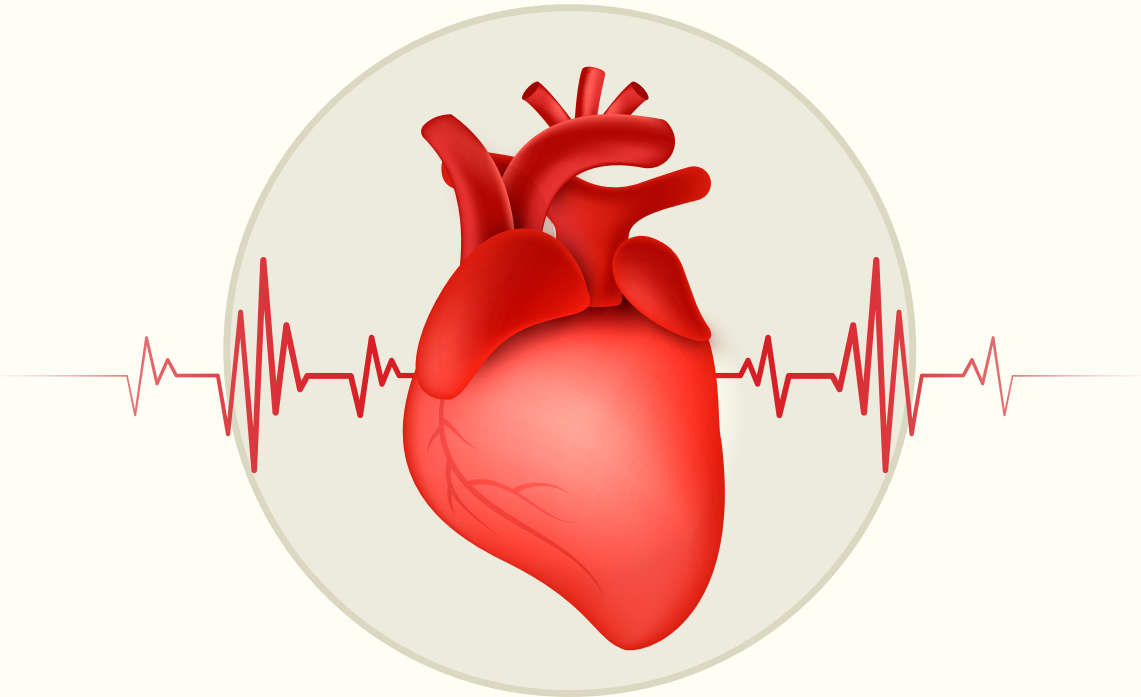

↓ **Sympathetic nervous activity**
LF/HF ratio ($p = 0.028$)
LF% ($p = 0.022$)

↑ **Parasympathetic activity**
HF% ($p = 0.062$)

More pronounced results in the addiction group

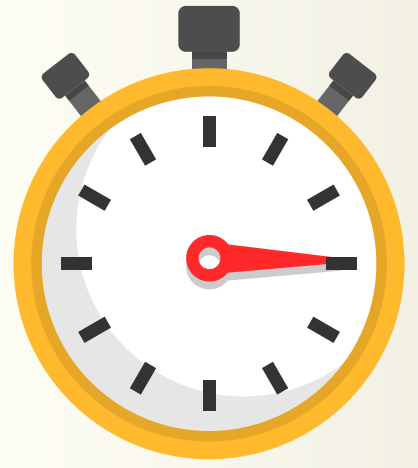

15 minutes cupping

↑
- Increased autonomic total power ($p = 0.010$)
- Improved sympathetic-parasympathetic modulation ($p = 0.011$)
- Enhanced parasympathetic activity ($p = 0.028$)

LF: low-frequency power; HF: high-frequency power

Dry cupping therapy can modulate autonomic activity and improve tissue compliance in a dose-dependent manner, with cupping at −300 mmHg negative pressure for 15 minutes, producing the most favorable outcomes
