# Supplementary material for: Duration-dependent effects of dry cupping on autonomic modulation and trapezius tissue compliance in smartphone users: a dose-finding study
Source: Front Physiol. 2026 Aug 3;17:1812427. doi: 10.3389/fphys.2026.1812427 (PMC13477954; doi:10.3389/fphys.2026.1812427)
Supplement: Supplementary file 2 [file Table1.docx]

**Supplementary** **Table S1**. Main effects and interaction effects of pressure, duration, and time on heart rate variability parameters.

| HRV | Group | Control (N = 13) | | Addiction (N = 19) | | p (F) value | |
| --- | --- | --- | --- | --- | --- | --- | --- |
|  | Trials | Before cupping | The end of cupping | Before cupping | The end of cupping | Time × Trial | Pressure × Duration × Time |
| TP  [ln(ms^2^)] | A | 7.10 ± 0.16 | 6.95 ± 0.19 | 7.58 ± 0.13 | 7.48 ± 0.15 | 0.010* | 0.178 |
|  | B | 7.01 ± 0.18 | 7.01 ± 0.19 | 7.36 ± 0.15 | 7.43 ± 0.16 | (3.982) | (1.900) |
|  | C | 7.25 ± 0.19 | 7.43 ± 0.17 | 7.30 ± 0.16 | 7.55 ± 0.14 |  |  |
|  | D | 7.16 ± 0.24 | 7.54 ± 0.23 | 7.41 ± 0.19 | 7.45 ± 0.19 |  |  |
| LF  [ln(ms^2^)] | A | 6.47 ± 0.17 | 6.33 ± .21 | 6.86 ± 0.14 | 6.68 ± 0.17 | 0.011* | 0.092 |
|  | B | 6.38 ± 0.19 | 6.34 ± 0.20 | 6.68 ± 0.16 | 6.65 ± 0.16 | (3.894) | (3.030) |
|  | C | 6.55 ± 0.21 | 6.75 ± 0.18 | 6.56 ± 0.17 | 6.78 ± 0.15 |  |  |
|  | D | 6.57 ± 0.26 | 6.82 ± 0.24 | 6.71 ± 0.21 | 6.67 ± 0.19 |  |  |
| HF  [ln(ms^2^)] | A | 5.29 ± 0.22 | 5.06 ± 0.24 | 5.79 ± 0.18 | 5.84 ± 0.20 | 0.028* | 0.489 |
|  | B | 5.27 ± 0.23 | 5.36 ± 0.23 | 5.64 ± 0.19 | 5.83 ± 0.19 | (3.176) | (0.490) |
|  | C | 5.49 ± 0.22 | 5.66 ± 0.17 | 5.61 ± 0.18 | 5.79 ± 0.14 |  |  |
|  | D | 5.28 ± 0.26 | 5.63 ± 0.25 | 5.58 ± 0.22 | 5.81 ± 0.20 |  |  |
| LF/HF  ratio | A | 1.17 ± 0.19 | 1.27 ± 0.22 | 1.07 ± 0.16 | 0.84 ± 0.18 | 0.219 | 0.348 |
|  | B | 1.11 ± 0.19 | 0.98 ± 0.19 | 1.04 ± 0.16 | 0.82 ± 0.15 | (1.504) | (0.908) |
|  | C | 1.06 ± 0.20 | 1.09 ± 0.19 | 0.94 ± 0.17 | 0.99 ± 0.16 |  |  |
|  | D | 1.30 ± 0.16 | 1.20 ± 0.21 | 1.13 ± 0.13 | 0.86 ± .17 |  |  |
| LF% | A | 72.9 ± 3.7 | 73.6 ± 4.2 | 69.8 ± 3.1 | 66.1 ± 3.5 | 0.362 | 0.447 |
|  | B | 70.8 ± 3.9 | 68.8 ± 3.9 | 69.7 ± 3.2 | 65.4 ± 3.2 | (1.079) | (0.593) |
|  | C | 71.5 ± 4.2 | 71.4 ± 3.6 | 67.5 ± 3.5 | 67.8 ± 3.0 |  |  |
|  | D | 75.6 ± 3.0 | 73.7 ± 4.2 | 71.9 ± 2.5 | 67.0 ± 3.4 |  |  |
| HF% | A | 19.5 ± 2.8 | 18.8 ± 3.6 | 21.3 ± 2.3 | 25.4 ± 2.9 | 0.471 | 0.173 |
|  | B | 21.3 ± 3.0 | 22.1 ± 3.2 | 22.3 ± 2.4 | 24.2 ± 2.6 | (0.849) | (1.942) |
|  | C | 21.2 ± 3.0 | 19.9 ± 2.7 | 22.9 ± 2.5 | 22.5 ± 2.2 |  |  |
|  | D | 19.0 ± 2.3 | 19.0 ± 3.7 | 20.3 ± 1.9 | 24.4 ± 3.0 |  |  |

HRV, heart rate variability; TP, total power; LF, low-frequency power; HF, high-frequency power; LF/HF, LF to HF ratio; LF%, LF in normalized units. Trials: A, ˗300 mmHg + 5 min; B, ˗450 mmHg + 5 min; C, ˗300 mmHg + 15 min; D, ˗450 mmHg + 15 min.

Data are presented as mean ± SEM; *p < 0.05
